# Novel anti-CD38 humanized mAb SG003 possessed enhanced cytotoxicity in lymphoma than Daratumumab via antibody-dependent cell-mediated cytotoxicity

**DOI:** 10.1186/s12896-019-0524-8

**Published:** 2019-05-22

**Authors:** Tao Yu, Chunxia Qiao, Ming Lv, Luqun Tang

**Affiliations:** 1 Department of Oncology, the First Affiliated Hospital of Nanjing Medical University, Nanjing, 210029 China; 2 State key Laboratory of Toxicology and Medical Countermeasures, Beijing Institute of Pharmacology and Toxicology, 27 Taiping Road, Beijing, 100850 China; 3 Sumgen Biotech co., Ltd., Hangzhou, 310000 China; 4 Department of Radiation Oncology, the Second Affiliated Hospital of Nanjing Medical University, Nanjing, 210011 China

**Keywords:** CD38, Daratumumab, Monoclonal antibody, SDR-grafted humanization

## Abstract

**Background:**

In vivo use of monoclonal antibodies has become routine clinical practice in the treatment of human cancer. CD38 is an attractive target, because it has double roles, as a receptor and an ectoenzyme. Daratumumab, an anti-CD38 antibody, is currently in the clinical trials for multiple myeloma.

**Results:**

Here we obtained a humanized anti-CD38 antibody, SG003, using SDR-grafting method. SG003 possessed stronger antigen binding activity than Daratumumab, and its epitope was far from that of Daratumumab, an anti-CD38 antibody currently in the clinical trials for multiple myeloma; besides, SG003 showed enhanced antibody-dependent cell-mediated cytotoxicity function and in vivo inhibitory efficacy of tumor growth in xenograft mice model.

**Conclusion:**

SG003 seemed to be a good option to improve the curative effect of CD38-related cancers.

## Background

The majority of therapeutic strategies for cancer treatment targets surface molecules expressed by solid tumors or leukemic cells, e.g. erbB2, and CD20, etc., one of which is human CD38. CD38 is a 46-kDa type II trans-membrane glycoprotein with a long 256AA extracellular domain and a short 20AA N-terminal cytoplasmic tail. Functions of CD38 include receptor-mediated activation and ectoenzymatic activities that contribute to intracellular calcium mobilization. CD38 is defined originally as a T-cell activation molecule. Under normal conditions, it’s expressed highly in committed progenitor bone marrow (early BM cells are CD38 negative), and B lymphocytes in germinal centers. It is also expressed at low levels on lymphoid and myeloid cells and in some tissues of non-hematopoietic origin [1]; besides, many studies reported that myeloma cells express CD38 in the overwhelming majority of patients, although at varying surface densities. CD38 signals may operate on a myeloma background by modulating miRNAs. For example, among the miRNAs downregulated by CD38 ligation, miR-193b functions as a tumor suppressor miRNA [2]. In a word, its high expression on the cells in multiple myeloma (MM) [3] suggest it a potential therapeutic monoclonal antibody (mAb) target in MM.

By now, several effective anti-human CD38 mAbs have been generated against several forms of human CD38+ cancers such as MM [4–6]. Daratumumab, a human mAb, is a first-class anti-CD38 therapeutic antibody approved by FDA for the treatment of relapsed multiple myeloma in 2015 that represents unique cytotoxic activities [7]. In SCID mouse xenograft tumor model, Daratumumab was active even at low concentrations [7]. CDC function was an important feature of Daratumumab, while less of other anti-CD38 antibodies could kill tumor cells by CDC function. In addition to CDC, Daratumumab induced ADCC function only in some kinds of cancer cells, e.g. leukemia cells, or patient MM tumor cells. Nijhof et al. [8] reported that in bone marrow samples, Daratumumab induced both CDC and ADCC in vitro effectively, and there is a significant association between Daratumumab-induced CDC/ADCC and the expression level of CD38 in MM patients. Recently, Schutze et al. have generated a CD38-specific biparatopic heavy chain antibodies, which elicited CDC toward CD38-expressing myeloma cells more effectively than daratumumab [9].

The satisfactory function of Daratumumab in preclinical research led to its clinical trials. In the first phase 1/2 clinical trial, Daratumumab was administered as a single agent in relapsed/refractory myeloma patients. In the group of 16 mg/kg, the ORR (overall response rate) was 36%, and the DOR (median duration of response) was not reached; besides, the rate of PFS (progression-free survival) over 12-month reached up to 65% [10, 11]; then, in the phase II trial (SIRIUS), the median PFS of MM patients was 3.7 months, and 12-month OS (overall survival) rate was 64.8% [12]; In SIRIUS and GEN501 studies [13], the ORR was 31%, and the OS was about 19.9 months, demonstrating that Daratumumab monotherapy was beneficial in patients with pre-treated and/or relapsed/refractory myeloma [14, 15]; meanwhile, Daratumumab is also under investigation in combination with other MM regimens [16]; Besides, two additional anti-CD38 antibodies have also entered clinical trials for MM and other CD38+ hematologic malignancies, MOR202 [17, 18] and isatuximab (SAR650984) [19], that are being tested alone and in combination with standard therapy.

In chronic lymphocytic leukemia (CLL), CD38 is also one of the surface molecular markers for clinical use [20]. It is now generally accepted that CD38+ leukemia patients have a shorter progression-free interval, require earlier and more frequent treatments, and ultimately lower survival rate. Daratumumab was tested to have anti-tumor activity in leukemia [21, 22]. Recently, our team screened a mouse-anti-CD38 mAb, 3G3, by hybridoma technology, which showed good anti-leukemia activity in vitro. Here we humanized 3G3 to have a SDR-grafted antibody, SG003, whose affinity to bind to CD38 was higher than Daratumumab, and the epitope of SG003 was different from that of Daratumumab; moreover, SG003 showed stronger ADCC function and in vivo inhibitory efficacy of tumor growth in xenograft mice model, suggesting its potential to achieve improved curative effect in patients with leukemia.

## Results

### 3G3 can kill the lymphocytes by ADCC function

**Fig. 1:**
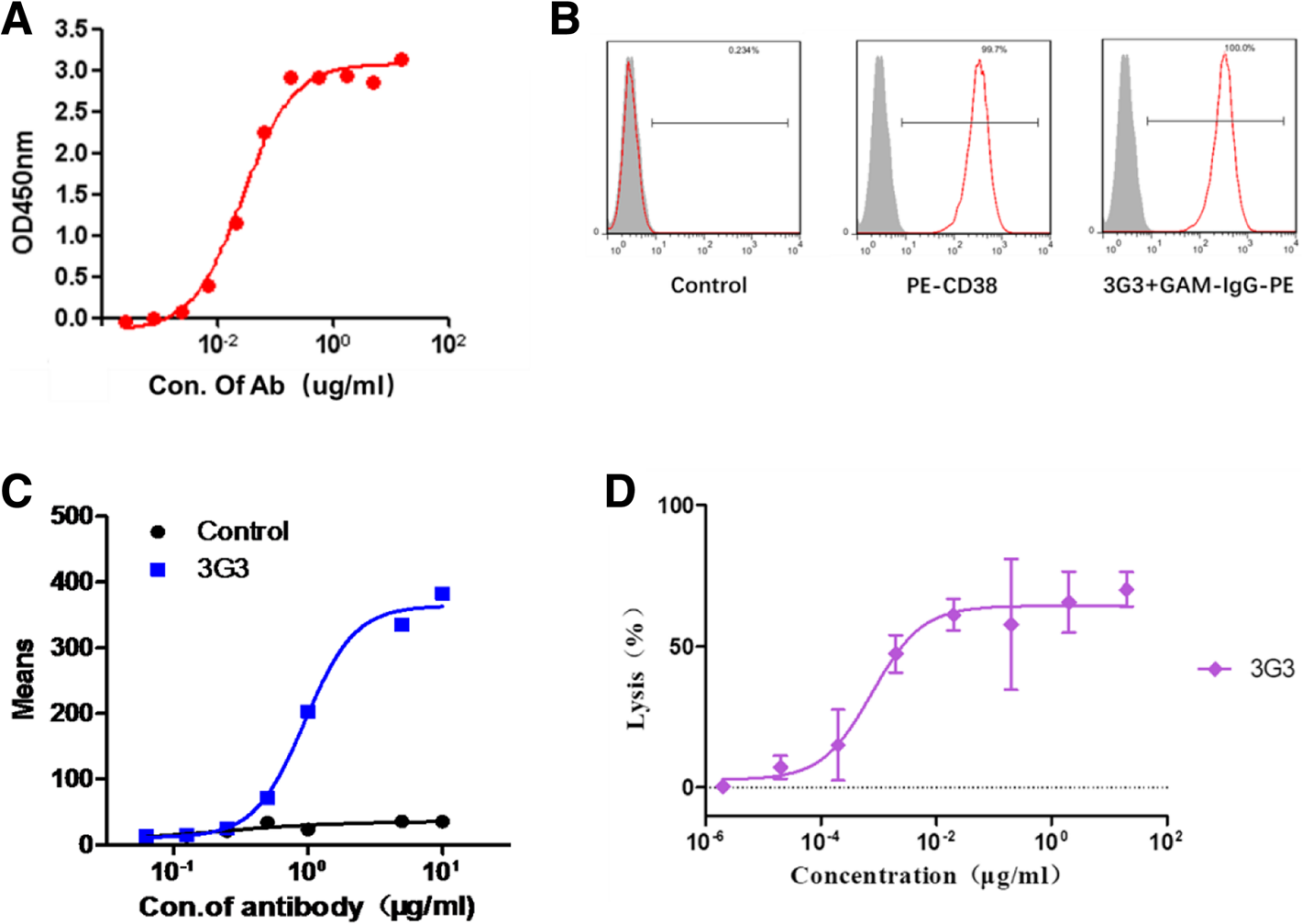
3G3 bound to the membrane CD38 to kill Raji cells by ADCC. **a** ELISA analysis of 3G3 to bind coated CD38. 3G3 bound to CD38 well on a dose-dependent manner; **b** and **c** 3G3 bound to extracellular CD38 on Raji cells (**b**) on a dose-dependent manner (**c**). Human IgG (polyclonal) was set as negative control; **d** ADCC function of 3G3 in Raji cells on a dose dependent manner with the EC_50_ value of ~ 5 ng/mL

3G3 was screened out using classical hybridoma technology. It bound to the antigen CD38 on a dose-dependent manner both by ELISA (Fig. 1a) and flow cytometry in Raji cells (Fig. 1b and c); meanwhile, 3G3 showed satisfactory cytotoxicity function on a dose-dependent manner. According to the ADCC results in Raji cells, the EC_50_ value of 3G3 was about 0.005 μg/mL (Fig. 1d).

### SG003 possessed stronger antigen binding activity than Daratumumab

**Fig. 2:**
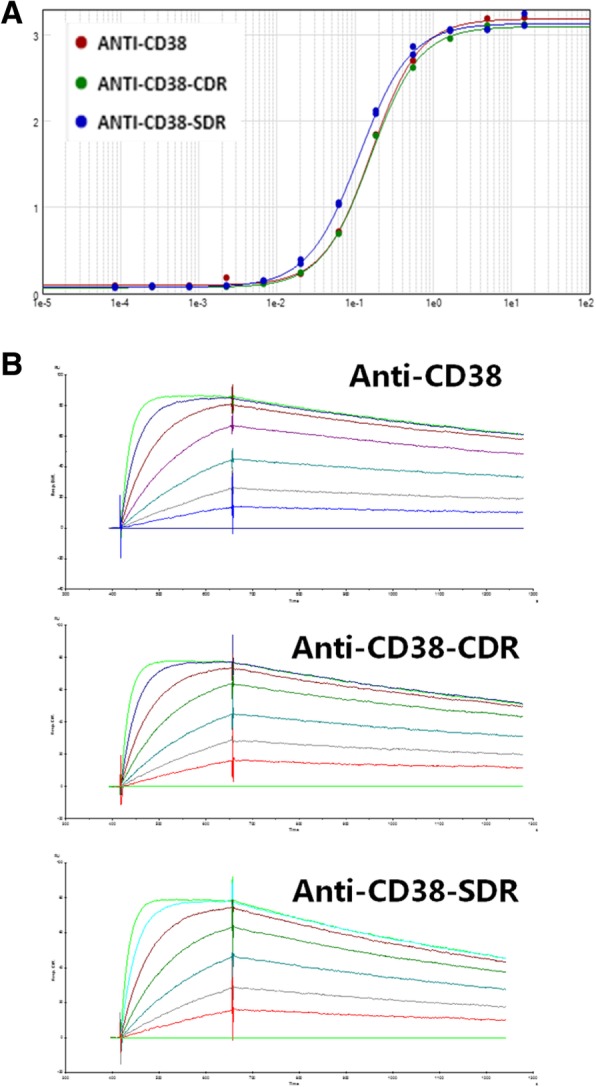
Identification of humanized anti-CD38 antibodies to bind the antigen. **a** ELISA analysis of the three antibodies to bind the coated CD38. Both CDR-grafted 3G3 (anti-CD38-CDR) and the SDR-grafted 3G3 (anti-CD38-SDR) showed similar antigen binding activity to 3G3 (anti-CD38) on a dose-dependent manner; **b** BiAcore analysis of the three antibodies to bind soluble CD38, showing that the SDR-grafted 3G3 possessed similar affinity to 3G3

3G3 was humanized using CDR- or SDR-grafting method, and SG003, which was obtained from SDR-grafting, was identified similar to 3G3 in CD38 binding. As shown in Fig. 2a, ELISA assay displayed satisfactory bio-function of both CDR-grafted 3G3 (anti-CD38-CDR) and SDR-grafted 3G3 (anti-CD38-SDR, also named SG003) to bind the soluble CD38 on a dose-dependent manner, which was similar to 3G3; furthermore, according to the BiAcore analysis data, SG003 possessed similar affinity to 3G3 (Fig. 2b), with the *Ka* and *Kd* value of 2.86*10^6^ 1/Ms. and 9.18*10^− 4^ 1/s, respectively (Table 1).

**Table 1:**
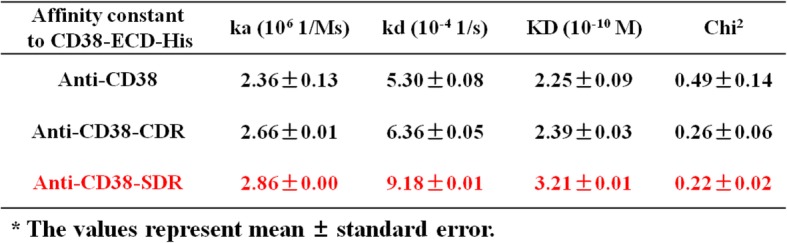
Affinity constant of anti-CD38 antibodies. The SDR-grafted 3G3 (Anti-CD38-SDR) had similar/a little bit higher Ka and Kd value to 3G3, therefore its affinity constant is similar to 3G3

^*^The values represent mean ± standard error

**Fig. 3:**
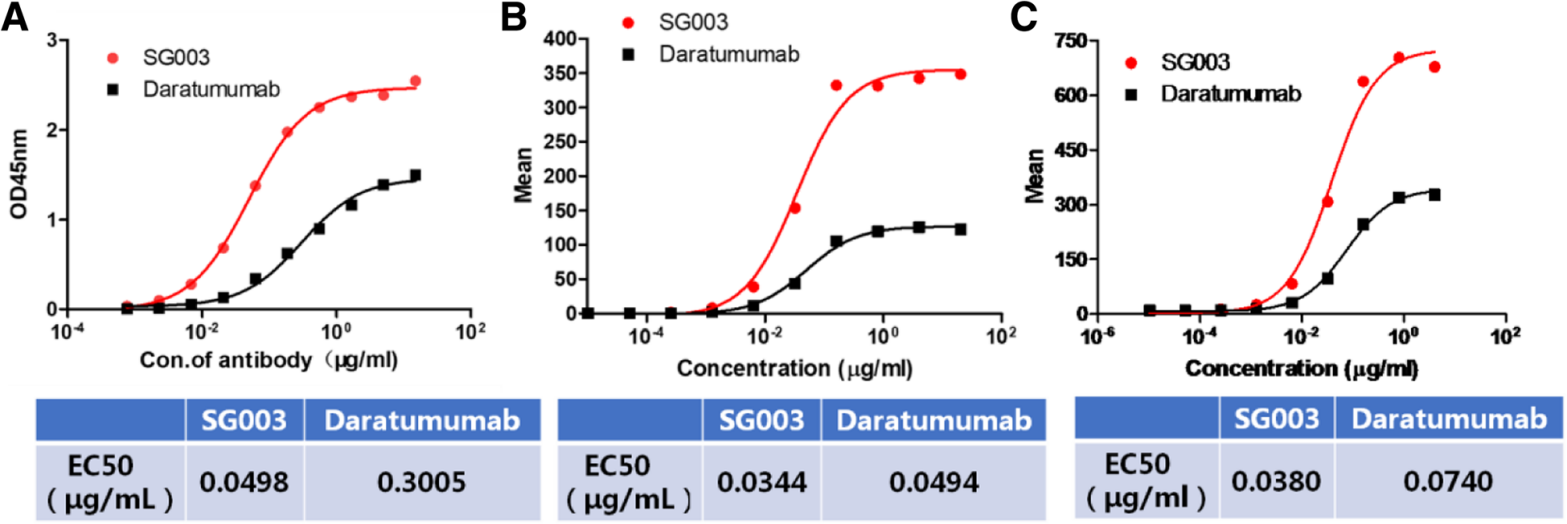
SG003 (the SDR-grafted 3G3, Anti-CD38-SDR) possessed stronger antigen binding activity than Daratumumab. ELISA analysis (**a**) showed the EC_50_ value of SG003 to bind coated CD38 (0.0498 μg/mL), which is ~ 6 times stronger than that of Daratumumab (EC_50_ = 0.3005 μg/mL), while the maximum OD value of SG003 was also higher obviously than Daratumumab; Similarly, in either Raji or Daudi cells, flow cytometry assays also showed stronger activity of SG003 than Daratumumab to bind membrane CD38, for the EC_50_ value was 0.0344 or 0.0380 μg/mL in SG003-treated Raji (**b**) or Daudi (**c**) cells, and more importantly, the mean value was also much higher in SG003-bound cells, suggesting that SG003 might induce stronger tumor killing effect

**Fig. 4:**
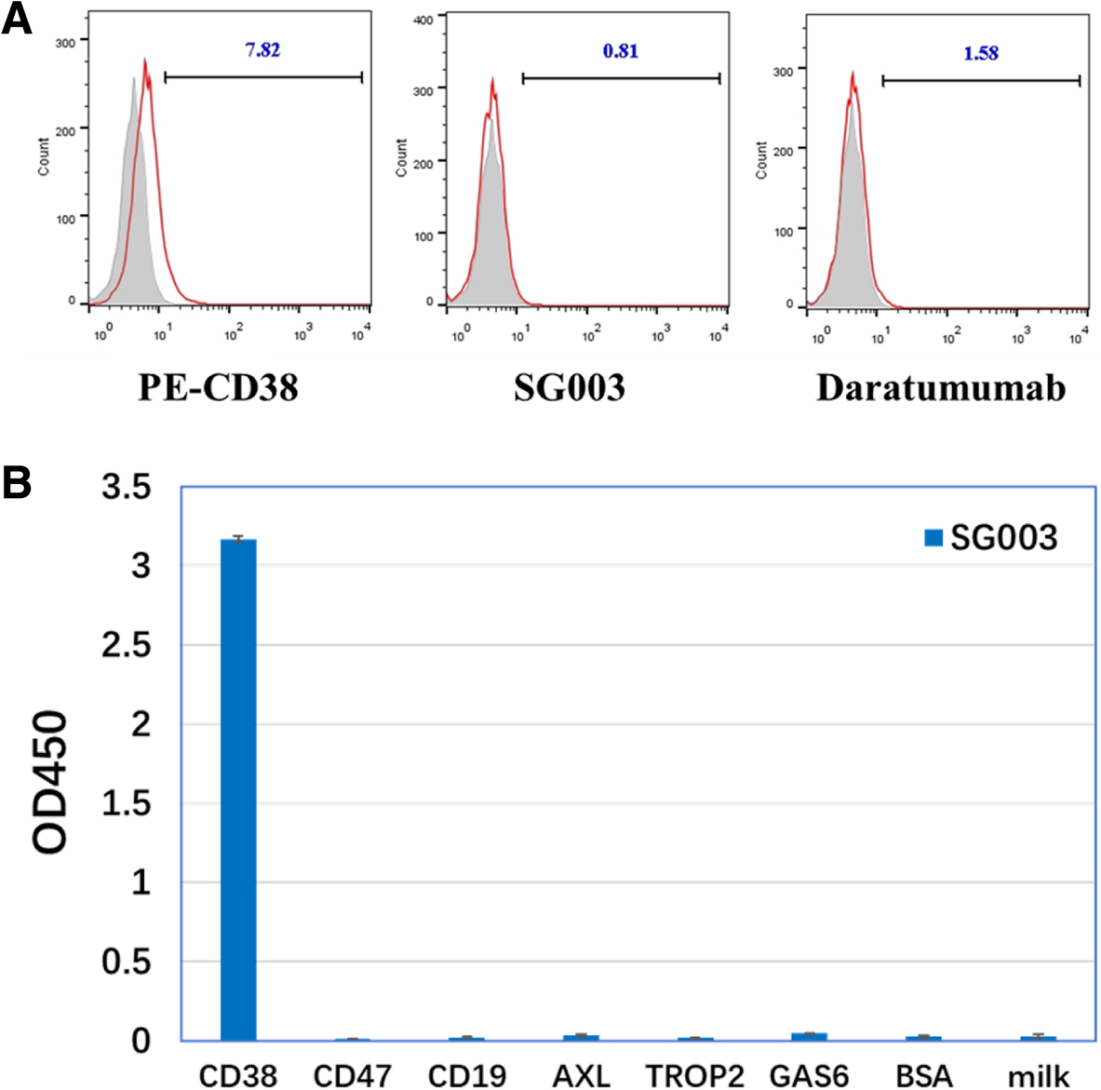
SG003 had no/weak cross-reactivity to a series of glycoproteins. **a** flow cytometry analysis of SG003 to bind 293 T cells (CD38 negative); **b** SG003 didn’t bind to the extracellular domain of many receptors, e.g. CD47, CD19, BSA, etc. by ELISA detection

Meanwhile, we compared SG003 with the first-in-class anti-CD38 therapeutic antibody Daratumumab. Dosage ELISA showed the EC_50_ value of SG003 as 0.0498 μg/mL to bind the coated sCD38, which is ~ 6 times stronger than Daratumumab (EC_50_ = 0.3005 μg/mL); moreover, the maximum OD value of SG003 was obviously higher than Daratumumab (Fig. 3a, 2.4 of SG003 V.S. 1.5 of Daratumumab); Similarly, flow cytometry assays also showed that SG003 had stronger cell surface CD38-binding activity than Daratumumab in either Raji (Fig. 3b) or Daudi (Fig. 3c) cells. More importantly, the mean value of SG003 group was much higher, suggesting that SG003 might bound to the extracellular domain of CD38 in vivo*,* thus possibly induce stronger cytotoxicity. More importantly, SG003 had no/weak cross-reactivity to bind CD38- tumor cells (e.g. 293 T) by flow cytometry detection (Fig. 4a), or a series of extracellular domain of many receptors/glycol-proteins (e.g. CD47, CD19, AXL, TROP2, GAS6, etc.) by ELISA detection (Fig. 4b).

### The epitope of SG003 was different from that of Daratumumab

**Fig. 5:**
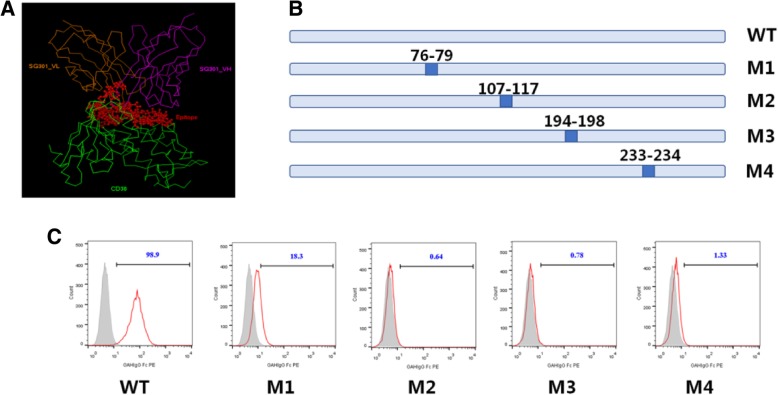
The epitope of SG003 was different from Daratumumab. **a** theoretical structure of the extracellular domain of CD38 and SG003 complex, according to which the potential epitope of SG003 located in ^76^E^78^R^79^H^107^Q^111^K^114^T^115^Q^116^T^117^V^194^R^195^R^198^E^233^E^234^K in CD38; **b** CD38 and its four mutations. The indicated sites were replaced with alanine; **c** Flow cytometry analysis of SG003 to bind CD38 mutations. The replacement of residues 76–79, 107–117, 194–198, or 233–234 induced the failure of SG003 to recognize CD38, while the epitope of Daratumumab were 233–246 and 267–280 of CD38

**Fig. 6:**
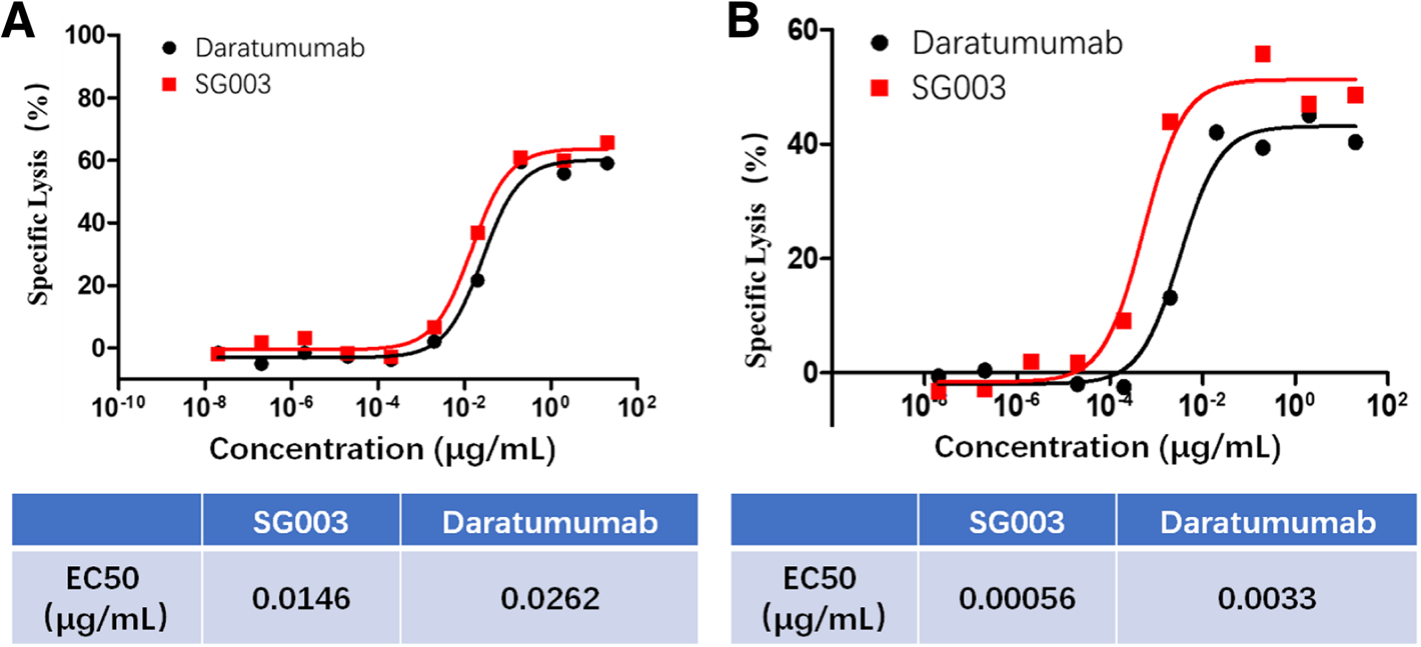
ADCC function of SG003 was stronger than Daratumumab. The EC_50_ value of SG003 was 0.0146 μg/mL in Raji (**a**), and 0.00056 μg/mL in Daudi cells (**b**), while in Daratumumab treated samples, the EC_50_ value was 0.0262 μg/mL and 0.0033 μg/mL, respectively. The unique epitope of SG003 might be beneficial to its stronger ADCC function

Using antibody homological modeling, molecular docking, and dynamic simulation methods, we constructed the theoretical structure of CD38 and SG003 complex (Fig. 5a), according to which the potential epitope of SG003 located in ^76^E^78^R^79^H^107^Q^111^K^114^T^115^Q^116^T^117^V^194^R^195^R^198^E^233^E^234^K of CD38. Then, a series of CD38 mutants were designed using alanine replacement (Fig. 5b) and displayed on 293 T cell surface method. As shown in Fig. 5c, SG003 couldn’t bind to these mutants, confirming the importance of residues 76–79, 107–117, 194–198 and 233–234 to recognize SG003. Meanwhile, the epitope of Daratumumab was 233–246 and 267–280 of CD38 [7], which was far from that of SG003; Moreover, in ADCC assays, the EC_50_ value of SG003 was 0.0146 μg/mL in Raji (Fig. 6a), and 0.00056 μg/mL in Daudi cells (Fig. 6b), both of which were lower than that of Daratumumab (0.0262 and 0.0033 μg/mL, respectively). We inferred SG003’s epitope might contribute to its stronger ADCC function, even though the mechanism was unknown to need further study.

### SG003 inhibits the growth of xenograft tumor in SCID mice

**Fig. 7:**
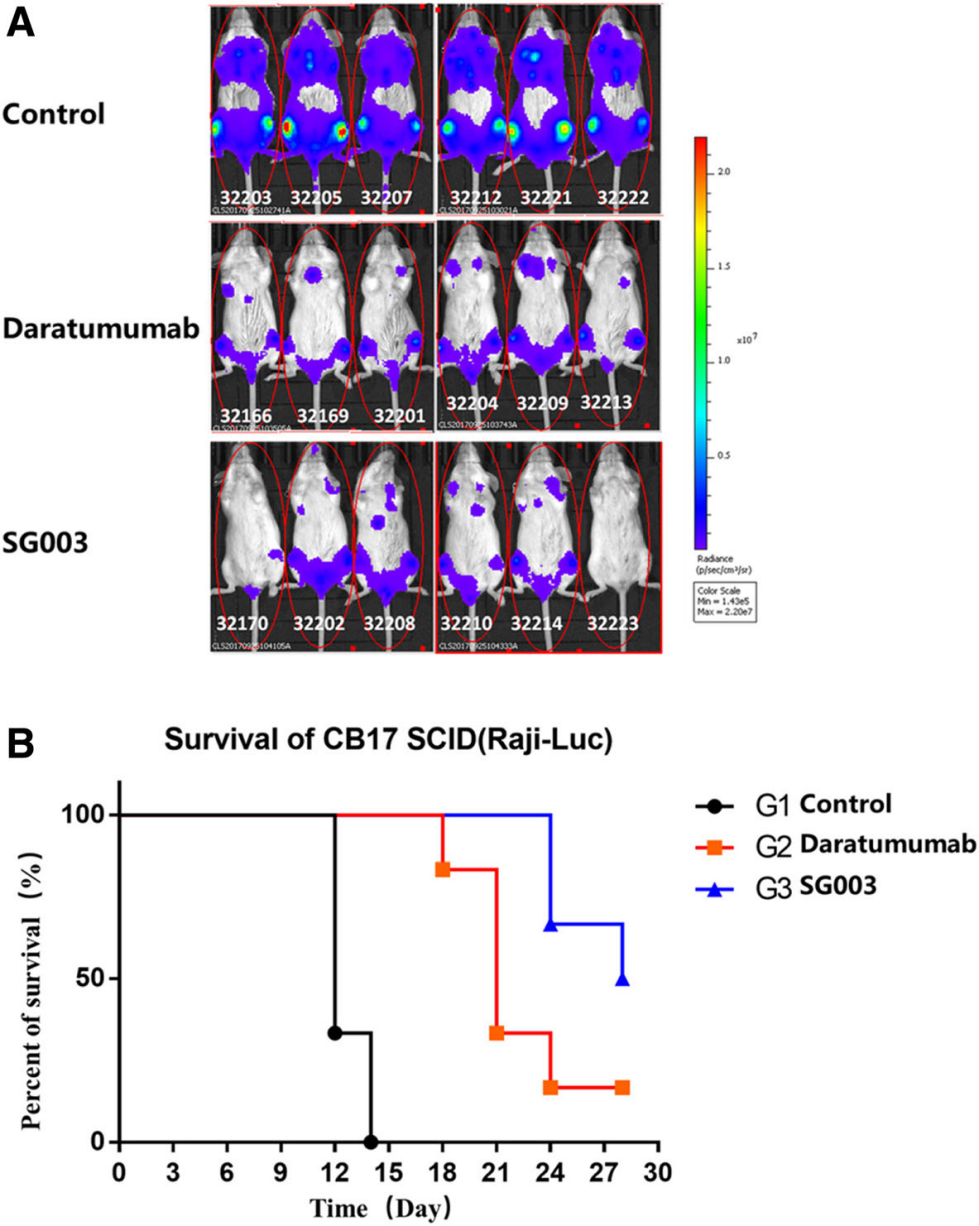
SG003 inhibits outgrowth of CD38-expressing Raji xenografts in SCID mice model. **a** Light emission of mice from the back side. SCID mice were inoculated with luciferase-expressing Raji cells, and 100 μg/mouse SG003 was injected to inhibit the tumor growth. Mice infused with Daratumumab or human IgG (100 μg/mouse) were set as positive or negative controls. **b** Survival Rate of Raji xenograft mice (*n* = 6). The experiments were done twice SG003 had satisfactory anti-tumor activity in vivo. 3 mice in SG003 group were alive at the endpoint, which was obviously higher than control group (G1, all dead), and even the Daratumumab-treated group (G2, 1 mouse alive)

Prior to cell injection, SCID mice were healthy with the body weight of 17~18 g/mouse. Mice were injected subcutaneously with CD38+ Raji cells. After ~ 7 days, SG003 were divided into 3 groups (*n* = 6) and inoculated *i.v.* with Daratumumab, SG003 or isotype control. Figure 7a showed the light emission of mice from the back side. The two groups of antibody-treated mice carried much smaller lump than the control group; more interestingly, SG003 seemed to inhibit the growth of Raji xenografts similar to Daratumumab. We were pleased to see that 2/6 mice had relapsed tumor in SG003 group (mice number 32170 and 32,223); besides, the survival rate also testified SG003’s stronger anti-tumor activity in vivo (G3 group) ---- 3/6 mice survived at the endpoint, which was higher than the control group (G1, all mice were dead) and even the Daratumumab-treated mice (G2, 5/6 mice dead) (Fig. 7b), suggesting SG003 might have better potential in in vivo cancer therapy.

## Discussion

CD38 is a type 2 transmembrane protein, and its molecular weight (MW) is approximately 45 kDa. CD38 is a single chain protein, however, its functional structure is a dimer or a multimer [23–25]. CD38 can help synthesize the ADP-ribose from NAD to increase the cytoplasmic Ca2+ concentration. CD38 does not bind to the BCR complex, and it tends to aggregate in discrete membrane lipid micro domains to BCR in CLL cells [21]. CD38 also exert the same membrane distribution for TCR on T lymphocytes and CD16 on NK cells [26, 27]. Besides, CD38 can induce the phosphorylation of the intracellular substrate cascades to trigger the cytokine secretion and/or T lymphocyte proliferation [28].

CD38 is used as a disease marker for leukemia and myeloma and it is considered a negative prognostic marker for CLL. In most of MM patients, CD38 was found positive and several anti-CD38 mAbs have been screened out to enter the clinical trials; in CLL cells, CD38 was also considered to form a complex network delivering survival signals, which made CD38 an attractive target for CLL therapy.

Daratumumab that specifically binds to CD38 was the first therapeutic anti-CD38 drug approved by FDA for patients with MM [10, 12]. Daratumumab was shown to facilitate cell killing in various hematological tumors mainly via CDC, ADCC [7], and/or apoptosis upon secondary cross-linking [29]. It also induced the cytotoxicity of MM tumor cells in patient via CDC and ADCC to suggest its high anti-tumor activity *in vivo*.

In mouse model with the CD38+ CLL MEC2 cells and patient-derived xenografts (CLL-PDX), Daratumumab improved the overall survival rate of MEC2 mice significantly [22]. Besides, Homing of CLL cells to lymphoid organs is mainly coordinated by the CXCL12/CXCR4 axis. The effect of Daratumumab on CLL cells was demonstrated by inhibiting CXCL12-induced migration in vitro and interfering patient-derived CLL cells’ homing to spleen in NSG mice in vivo*.* Vaisitti et al. demonstrated CD38 together with CXCR4 controls homing of CLL cells because of CD38-CXCR4 interaction both in CLL primary cells and a xenograft mouse model [30]. Daratumumab also reduced CD49d mediated CLL cell adhesion by reducing MMP9 levels [22]. These unique and substantial effects of Daratumumab supported the possibility of anti-CD38 antibodies in clinical use of CLL patients.

Ghose et al. [31] have demonstrated that Daratumumab impairs multiple myeloma cell adhesion, which leads to an increased sensitivity of multiple myeloma to proteasome inhibition. Daratumumab was also shown to induce CDC in cancer cells from MM patients, similar to alemtuzumab (CD52 mAb), which has been found to kill B cell tumor cells mainly via CDC [32]. Interestingly, Daratumumab was demonstrated efficient to kill patient-derived CLL cells by ADCC in vitro, while exhibited negligible CDC in these cells [22], however, the reason why Daratumumab showed limited CDC activity in both primary CLL cells and CLL cell lines kept unknown. Here we screened a novel anti-CD38 antibody 3G3, which possessed enhanced cytotoxicity in B lymphocytes than Daratumumab via ADCC (Fig. 1). Then we humanized it to obtain SG003, a SDR-grafted mAb, whose affinity was similar to 3G3 (Fig. 2 & Table 1). We also noticed the predominant ADCC function triggered by SG003, which was much stronger than Daratumumab (Fig. 6), while its CDC function was too weak to be detected (data not shown).

Evidence suggest that the positioning of mAbs after binding to its antigen may be ideal for complement activation [33], and this may also exist in Daratumumab. In fact, induction of CDC was a very specific character of Daratumumab contrasting to other CD38 mAbs. Epitope mapping and site directed mutagenesis assays revealed its unique epitope, which might cluster and position its Fc region in a fine situation that permitted more optimal activation of complements. Here the affinity of SG003 was stronger than Daratumumab (Fig. 3), and its epitope was distinct (Fig. 5). We assumed that the distinct epitope of SG003 might contribute to its stronger ADCC but weaker CDC function in B lymphocytes as well as the stronger activity to inhibit the growth of xenografts *in vivo* and improve the survival rate of SCID mice (Fig. 7), although further studies should be done to confirm it.

## Conclusion

Contrasting to the first therapeutic anti-CD38 antibody Daratumumab, 3G3 possessed higher affinity, unique epitope, stronger ADCC function, as well as better in vivo curative effect. 3G3 seemed to be a good candidate with the potential to offer better therapeutic efficacy, especially in CD38+ leukemia patients.

## Methods

### Reagents

CD38 protein was purchased from Sino biological (Cat. No: 10818-H08H); Darzalex (Daratumumab) was purchased from Janssen Biotech; Human IgG Isotype control (Cat. No.: 02–7102) and HRP conjugated Goat anti-Human IgG (H + L) Secondary Antibody (Cat. No.: # A18805, dilution for ELISA: 1:2000) FROM Invitrogen; PE conjugated Goat anti-human polyclonal antibody (PE_GAH) were from eBiosciences (Cat. No.: 12–4998-82, Dilution for flow cytometry: 1:50); Dulbecco’s modified Eagle medium (DMEM) (Cat. No. 11965–092), RPMI 1640 (Cat. No. 11875–093), and fetal bovine serum (FBS) (Cat. No. 10438–034) were purchased from Thermo Fisher; DELFIA EuTDA Cytotoxicity Reagents was purchased from Perkin Elmer (Cat. No. AD0116); All other chemicals were obtained from commercial source of analytical grade.

### Cell lines

Human embryonic kidney epithelial cells 293 T (ATCC® CRL-3216™), B Lymphocyte Raji (ATCC®CCL-86™) and B Lymphoblast Daudi (ATCC®CCL-213™) were obtained from American Type Culture Collection (ATCC); Raji-luc lymphoma cells were generated as described previously to let Raji cells express luciferase [34, 35]; Human peripheral blood mononuclear cells (PBMC) were extracted from the whole blood using Ficoll-Paque Plus (GE Healthcare, Cat. No. 17–1440-02); 293 T were cultured in DMEM, while Raji and Daudi were cultured in RPMI-1640 (Gibco, Cat. No. 11875–093). Media was supplemented with 10% heat-inactivated FBS (Gibco, Cat. No. 26010–074) and 100 units/mL penicillin & streptomycin (Gibco, Cat. No. 15140–122). Cells were tested without mycoplasma contamination (LookOut Mycoplasma PCR detection kit, Cat. No. MP0035, Sigma-Aldrich, MO, USA) and cultured at 37 °C in 5% CO2.

### Antibody generation

Anti-CD38 Antibody 3G3 were generated by immunization of mice with recombinant CD38 protein. After isolation of mouse splenocytes and fusion, the hybridomas were tested for binding to CD38 by indirect ELISA, from which 3G3 was selected based on its high affinity and then its unique cytotoxicity ability of Raji cells. In all experiments, human IgG polyclonal antibody (pAb) was used as negative control.

According to the theoretical analysis, simulation and design, the VH/VL genes of CDR- or SDR- grafted 3G3 antibodies were obtained, then the humanized antibodies were prepared using our Flp-FRT mammalian system [36].

### ELISA

ELISA plates were coated with antigen (e.g. 2 μg/mL CD38 protein) at 4 °C overnight and then blocked with 1% BSA in PBS containing 0.05% Tween-20 for an hour at 37 °C. Diluted antibodies were added as first antibody and incubated for 2 hours at 37 °C. After washing, horseradish peroxidase conjugated goat anti-human IgG was added for an hour’s incubation at room temperature. Binding signals were visualized using TMB substrate and the light absorbance was measured with an ELISA reader at 450 nm.

### Cytotoxicity assay (ADCC)

ADCC activities of the antibodies were measured by DELFIA EuTDA Cytotoxicity Reagents (Perkin Elmer) according to the manufacturer’s instructions. Briefly, the tumor cells (target cells) were incubated with fluorescence-enhancing ligand, then incubated with the anti-CD38 antibodies for 1hat 37 °C, followed by the addition of the effector cells, human peripheral blood mononuclear cells (PBMC) (effector:target = 50:1). After an additional incubation for 4 h at 37 °C, the fluorescence of the supernatant was measured in a time-resolved fluorometer. Maximum release was determined by cell lysis in 0.2% Triton X-100. Percentage of lysis in each mAb-treated sample was calculated according to the following formula:

$$ \%\mathrm{Inhibition}=\left[\mathrm{experimental}\ \mathrm{release}-\mathrm{minimum}\ \mathrm{release}\right]/\left[\mathrm{maximum}\ \mathrm{release}-\mathrm{minimum}\ \mathrm{release}\right]\times 100. $$

### Computer-aided humanization and epitope prediction

The 3-D structure of CD38 or 3G3 were constructed by computer-guided homology modeling method using InsightII version 2005 (Molecular Simulations, San Diego, CA). According to the potential epitope of CD38, the 3-D complex structure of CD38 and 3G3 was modeled with molecular docking method and optimized using the modules Discover and CHARMM in InsightII. The non-bonded cutoff was 10 Å, non-bonded parameters and atomic charges were taken as defaults. A distance dependent dielectric constant was used as in vacuo calculations. The model was minimized using steepest descent (2000 steps) and conjugate gradient (5000 steps) methods, respectively.

Based on the complex structure, the six CDRs of 3G3 were kept while the FR regions were replaced to human sites, which offered CDR-grafted 3G3; meanwhile, the core sites of 3G3-CDRs were kept, and the FR regions as well as both ends of six CDRs were replaced to human residues, giving birth to SDR-grafted 3G3.

According to the theoretical structure of extracellular regions of human CD38 (CD38_ECD) and antibody (e.g. 3G3) complex, the potential epitopes were predicted, by which a series of CD38 mutants were designed.

### BIAcore

The antigen, commercial CD38 recombinant protein, was set as fluid phase. The parent antibody 3G3 was set as positive control, and the solvent (sample buffer) was set as negative control. The background was deducted according to the V-baseline in order to obtain the kinetic curves and calculate the dissociation constant (K_D_ value).

### Flow cytometry

Raji or Daudi cells were washed and then incubated with serial dilutions of mAbs or isotype IgG control. After washing and incubation with PE_GAH, samples were determined using BD FACS Calibur and the fluorescence density was analyzed with BD CellQuest Pro software. Experiments were performed on ice.

In epitope identification assay, using alanine replacement, a series of CD38 mutants were expressed on 293 T cell surface. Then cells were incubated with SG003 and then PE_GAH for detection.

### In vivo assays in mice

Eighteen female, 8- to 10-wk-old C.B-17 SCID mice (C.B-17/IcrCrl-scid-BR) were purchased from Beijing Vital River Laboratory Animal Technology Co., Ltd. and housed in filter-top cages (3 mice per cage) in SPF-level Facility fed with Sterilized food and water. During the animal assays, we followed the ARRIVE guidelines for the use of mice (https://www.nc3rs.org.uk/arrive-guidelines). We noticed animal ethics during the research with 3R principles (Replacement, Reduction and Refinement), and use or treatment of mice were in strict agreement with the guidelines for the care and use of research animals and approved by the Animal Ethics Committee of Beijing Institute of Pharmacology and Toxicology (Ethic permission number: PTAE2017010). Mice were checked at least three times a week for signs of discomfort and for general appearance. After the study, the mice were sacrificed using anesthesia by *i.p.* injection of 80 mg/kg ketamine, 20 mg/kg xylazine and 0.6 mg/kg atropine, and then the cervical dislocation method, and we used deep-freezing to confirm the death.

For the xenograft model, 2.5*10^6^ Raji-luc cells per mouse were injected *s.c..* In a week, mice were randomly divided into three groups, administered *i.v.* with 5 mg/kg of anti-CD38 mAb or human IgG isotype control, and observed twice a week for 4 weeks after administration of antibody (*n* = 6). On day 28, mice were injected *i.p.* with synthetic D-luciferin (acid form) and luminescence was measured. For imaging, mice were anesthetized by *i.p.* injection of 80 mg/kg ketamine, 20 mg/kg xylazine, and 0.6 mg/kg atropine mixture before D-luciferin was given in 2.5 mg/200 μl 10 mM Tris base/mouse. Imaging of each mouse was done using a VersArray 1300B device detector (Roper Scientific, Vianen, Netherlands) and photons emitted from the luciferase system were counted after an exposure period of 5 min. The experiments were done twice. MetaVue software (purchased from Molecular Devices) was used for data collection and analysis.

### Statistical analysis

All experiments were done at least two times. Data are expressed as mean ± SD. Pair wise differences between several groups were compared. Statistical analysis was performed by Student test or by repeated measures one-way ANOVA. Treatments were taken as statistically significant when *P*-values of less than 0.05.

## Abbreviations

ADCC: Antibody-dependent cell cytotoxicity
ATCC: American Type Culture Collection
BCR: B cell receptor
BM: Bone marrow
CDC: Complement-dependent cytotoxicity
DMEM: Dulbecco’s modified Eagle medium
DOR: Median duration of response
FBS: Fetal bovine serum
mAb: Monoclonal antibody
MM: Multiple myeloma
MW: Molecular weight
ORR: Overall response rate
OS: Overall survival
pAb: Polyclonal antibody
PBMC: Peripheral blood mononuclear cells
PFS: Progression-free survival
